# Growing Tungsten Nanophases on Carbon Spheres Doped with Nitrogen. Behaviour as Electro-Catalysts for Oxygen Reduction Reaction

**DOI:** 10.3390/ma14247716

**Published:** 2021-12-14

**Authors:** Teresa Briz-Amate, Jesica Castelo-Quibén, Esther Bailón-García, Abdalla Abdelwahab, Francisco Carrasco-Marín, Agustín F. Pérez-Cadenas

**Affiliations:** 1 Carbon Materials Research Group, Department of Inorganic Chemistry, Faculty of Sciences, University of Granada, Avenida de Fuente Nueva s/n, 18071 Granada, Spain; teresabriz@correo.ugr.es (T.B.-A.); jesicacastelo@ugr.es (J.C.-Q.); fmarin@ugr.es (F.C.-M.); afperez@ugr.es (A.F.P.-C.); 2 Unit of Excellence in Chemistry Applied to Biomedicine and the Environment, University of Granada, Avenida de Fuente Nueva s/n, 18071 Granada, Spain; 3 Materials Science and Nanotechnology Department, Faculty of Postgraduate Studies for Advanced Sciences, Beni-Suef University, Beni-Suef 62511, Egypt; aabdelwahab@psas.bsu.edu.eg; 4 Faculty of Science, Galala University, Suez 43511, Egypt

**Keywords:** carbon nanospheres, tungsten carbide, carbon–tungsten composites, nitrogen-doped electro-catalysts, oxygen reduction reaction

## Abstract

This work shows the preparation of carbon nanospheres with a high superficial nitrogen content (7 wt.%), obtained by a simple hydrothermal method, from pyrocatechol and formaldehyde, around which tungsten nanophases have been formed. One of these nanophases is tungsten carbide, whose electro-catalytic behavior in the ORR has been evaluated together with the presence of nitrogen surface groups. Both current and potential kinetic density values improve considerably with the presence of tungsten, despite the significant nitrogen loss detected during the carbonization treatment. However, the synergetic effect that the WC has with other electro-catalytic metals in this reaction cannot be easily evaluated with the nitrogen in these materials, since both contents vary in opposite ways. Nevertheless, all the prepared materials carried out oxygen electro-reduction by a mixed pathway of two and four electrons, showing remarkable electro-catalytic behavior.

## 1. Introduction

Taking into account the energy and environmental crisis that we are suffering from nowadays, there is a growing interest in finding new sources of energy that respect the environment, especially for applications in the transport sector. The development of electric vehicles is one of the most promising alternatives to replace combustion engines nowadays. Therefore, the production of electrical energy from chemical reactions, through the use of fuel cells, is a really interesting field from the industrial and fundamental research point of view [1,2,3,4,5,6]. The oxygen reduction reaction (ORR) is the reaction that takes place at the cathode of a fuel cell. In the literature published to date, several works can be found about the synthesis and optimization of electro-catalytic materials for this reaction [7,8,9,10,11,12].

Among the published works, platinum-based electro-catalysts are the most studied, since Pt is the most active metal for the ORR. However, the increase in the price of platinum and other precious metals, such as Pd or Ir, makes it more difficult to market the devices that contain them. This is the reason why non-precious metal electro-catalysts are more numerous and studied to reduce the costs of fuel cells [8,13,14,15,16,17].

In this regard, tungsten-based electro-catalysts have received great interest recently [18,19,20,21,22,23,24,25]; for example, it has been suggested that tungsten carbide can improve the activity and dispersion of supported Pt [19,22,26,27]. Although some works that have developed electro-catalysts based on tungsten carbide doped with iron and nitrogen showed an indirect pathway for the ORR (two-electron pathway) [28], others have been shown to work via four-electron pathways [21,24]. Fe/Co/WC @ NC hybrid catalysts have also been synthesized, consisting of uniform Fe_3_C and Co_3_C nanoparticles encapsulated in graphitized carbon, doped with nitrogen on their surface, and, in turn, wrapped with tungsten carbide (WC) in the form of a plate that works as an efficient catalyst for the oxygen reduction reaction (ORR) [29]. It was shown that the presence of WC promotes the ORR activity of Fe-/Cobased electro-catalysts. The results suggest that this composite exhibits efficient electro-catalytic activity, greater durability, and a greater capacity for methanol tolerance in comparison with commercial Pt/C catalysts for the ORR in an alkaline medium. These results are similar to those obtained with tungsten–cobalt carbides encapsulated in carbon (CoWC @ C) [30].

On the other hand, there are also some previous works using carbon gels doped with nitrogen as precursors of electro-catalysts based on tungsten carbide [25]. The obtained results were close to those obtained with a platinum catalyst in alkaline media, and even improved the onset potential. It should be noted that, in addition to the activity associated with tungsten carbide, the presence of mesoporosity also increases the electro-catalytic activity of these materials [21]. This work has implemented the development of doped carbon nanospheres with a notable nitrogen content, obtained by a simple hydrothermal method, to be coated with tungsten carbide, and to evaluate their electrocatalytic behavior in the ORR.

## 2. Materials and Methods

### 2.1. Synthesis of Materials

#### 2.1.1. Hydrothermal Synthesis of the Polymeric Spheres

Polymeric nanospheres were obtained by sol-gel hydrothermal synthesis using monomers of pyrocatechol and formaldehyde in an ethanol:water aqueous solution and in the presence of ammonia. The procedure carried out is an extension of the Stöber method [31], specifically following the procedure recently used by Moreno et al. [32].

Firstly, 0.212 g pyrocatechol (P) was dissolved in 60 mL of a water: EtOH solution (2.5:1 volume ratio). Following this, an NH_3_ and formaldehyde (F) solution was added, with the molar ratios NH_3_/P = 1.5 and F/P = 2. This mixture was placed into a 100 mL capacity autoclave (Parr Instruments, Moline, IL, USA) and then into an oven (memmert, Schwabach, Germany) at 100 °C for 24 h.

After 24 h the precipitate was filtered, washed with ethanol and the solvent was exchanged with acetone for 3 days, during which the acetone was changed daily. Finally, the material was dried under an IR lamp (Philips, Amsterdam, The Netherlands. The organic gel sphere sample thus obtained was named “S”.

#### 2.1.2. Impregnation with the Tungsten Precursor

The obtained organic gel spheres, S, were impregnated with an ammonium tungstate solution, [(NH_4_)_2_(WO_4_)], by the incipient impregnation method. The solution volume used was the porous V_TOTAL_ of S obtained by mercury intrusion porosimetry. The impregnation was carried out with solutions of ammonium tungstate of different concentrations to obtain doped spheres with 2%, 5% and 10% theoretical volumes of W in the final carbon. To do the calculation, a mass loss of 50% was assumed during the carbonization. Once impregnated, they were dried under an IR lamp overnight.

#### 2.1.3. Carbonization of the Polymeric Spheres and the Corresponding Obtainment of the Electro-Catalysts

Both undoped (S) and doped ammonium tungstate (SW2, SW5 and SW10) polymeric spheres were carbonized under a nitrogen atmosphere in a tubular oven at 900 °C with a heating rate of 2 °C·min^−1^. Once the tubular oven reached 900 °C, this temperature was maintained for 2 h. CS, CSW2, CSW5 and CSW10 samples were obtained following this procedure. It should be noted that the presence of NH_3_ in the synthesis, as well as the impregnation with ammonium tungstate, predicts functionalization with nitrogen in these materials.

### 2.2. Textural and Chemical Characterization

N_2_ adsorption–desorption at −196 °C was performed to analyze the porous texture. Before measuring the gas adsorption isotherms, the samples were outgassed overnight at 110 °C under high vacuum (10^−6^ mbar). The BET equation was applied to the N_2_ adsorption data to calculate the specific surface area S_BET_. The Dubinin–Radushkevich (DR) equation was also applied to the adsorption data in order to obtain the corresponding micropore volume (V_0_) and the micropore mean width (L_0_). Mesopore volume (V_MESO_) was calculated from the total pore volume obtained by the Gurvitch rule, subtracting the micropore volume obtained from the DR method.

The morphology of samples was analyzed by scanning electron microscopy (SEM) and high-resolution electron microscopy (HRTEM) using an FEI microscope model Quanta 400 (FEI, Hillsboro, OR, USA) and an FEI Titan G2 microscope (FEI, Hillsboro, OR, USA), respectively.

The crystalline phases of the obtained materials were analyzed by X-ray diffraction using a Bruker D8 Venture X-ray diffractometer (Bruker, Billerica, MA, USA) with Cu Kα radiation. The XRD patterns were recorded in the 2θ range from 6° to 77°. The average crystal size was estimated by the application of the Debye–Scherrer equation.

The surface chemistry was studied by X-ray photoelectron spectroscopy (XPS) using a Kratos Axis Ultra-DLD spectrometer (Kratos Analytical Ltd., Kyoto, Japan) equipped with a hemispherical electron analyzer connected to a detector DLD (delay-line detector) and an Al Kα monochromator with a power of 600 W. The X-ray source was a Mg/Al double anode with a power of 450 W. Binding energies were obtained with an accuracy of ±0.1 eV.

The total metal content was determined by thermogravimetric analysis (TGA) and inductively coupled plasma optical emission spectrometry (ICP-OES) using a TGA/DSC1 Thermogravimetric Analyzer from Mettler-Toledo (Mettler-Toledo International Inc, Greifensee, Switzerland), and an Optima 8300 ICP-OES from Perkin-Elmer (Waltham, MA, USA), respectively.

### 2.3. Electrochemical Measurements

All the electrochemical measurements were carried out in a three-electrode cell using the corresponding sample supported on the tip of the rotating disc electrode (RDE) as the working electrode.

Previously supporting every sample, the RDE tip, 0.071 cm^2^ in area, was carefully polished with alumina powder with 0.3 μm grain size.

To study the behavior of the materials in the absence and presence of oxygen, firstly, N_2_ was bubbled through the electrolytic solution (KOH 0.1M) until all the oxygen had been removed and cyclic voltammetry (CV) was performed from 0.4 V to −0.8 V with a sweeping rate of 50 mV·s^−1^ at 1000 rpm. The same experiment was accomplished with the saturated oxygen solution.

To study the mechanism and parameters of the ORR, linear sweep voltametries (LSV) were performed with a potential sweeping speed of 5 mV·s^−1^ at different rotation rates from 500 rpm to 4000 rpm, and in every case the electrolytic solution was saturated with oxygen. The data of these experiments were adjusted to the Koutecky–Levich equation to obtain the number of electrons transferred, as well as the kinetic density current (j_k_) and the ONSET potential, which the ORR starts.

## 3. Results and Discussion

**Figure 1:**
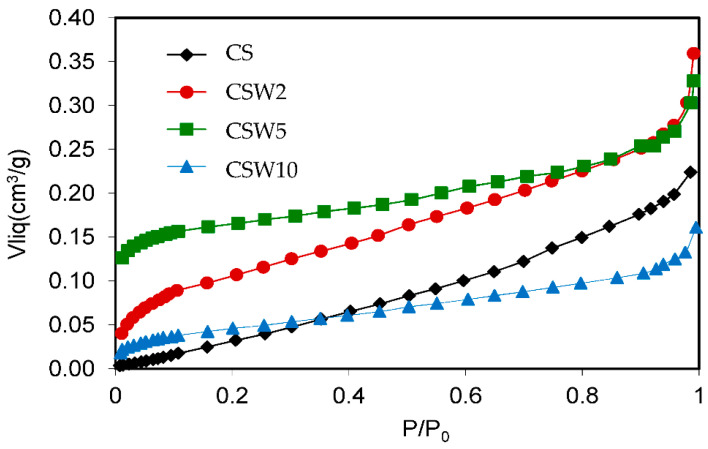
N_2_ adsorption isotherms.

The analysis of the porosity and surface area of the materials was achieved by physical adsorption of N_2_ at −196 °C, applying BET and Dubinin–Radushkevich (DR) methods. The mesopore volume (V_MESO_) was calculated from the total pore volume obtained by the Gurvitch rule, subtracting the micropore volume obtained from the DR method. The results are presented in Figure 1 and Table 1, which include the apparent surface area (S_BET_), the micropores volume (V_0_), the micropores average size (L_0_), the mesopore volumes, and the total contents of nitrogen and tungsten. Figure 1 shows how the carbon spheres (CS) are non-microporous materials, and the presence of a certain mesoporosity and/or interparticular spaces of the mesoporous order from their adsorption isotherm can be deduced. On the other hand, all the tungsten-coated spheres show type I–type IV hybrid isotherms, typical of mesoporous solids, but also with some adsorption at very low relative pressures, P/P_0_, revealing the presence of micropores.

**Figure 2:**
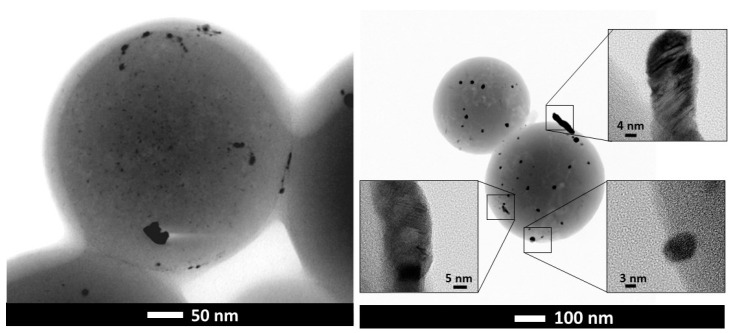
TEM and HRTEM images of CSW2.

**Figure 3:**
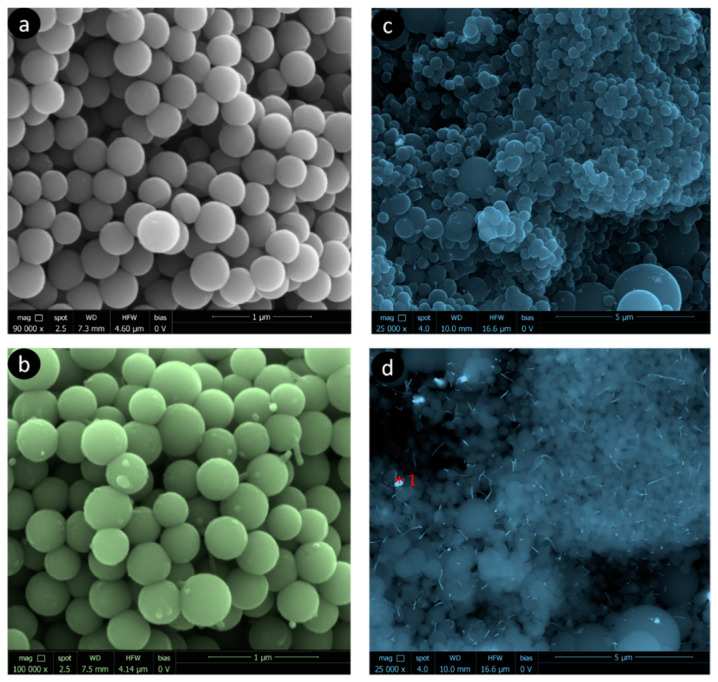
SEM images (**a**) CS; (**b**) CSW5; (**c**) CSW10 with ETD detector and (**d**) CSW10 with CBS detector.

**Figure 4:**
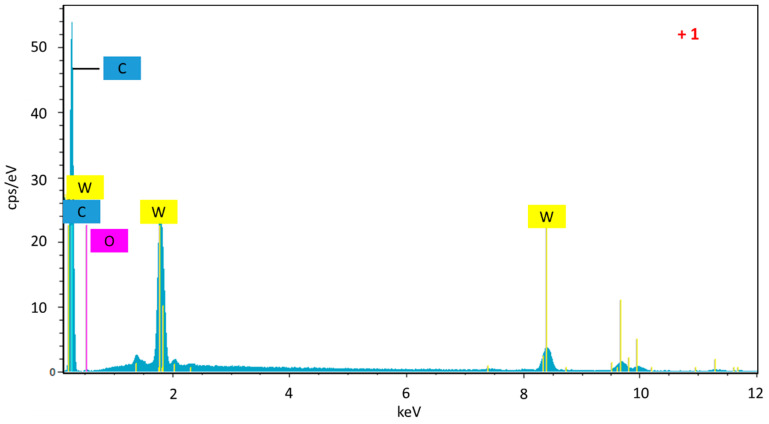
EDX analysis of the CSW10.

**Figure 5:**
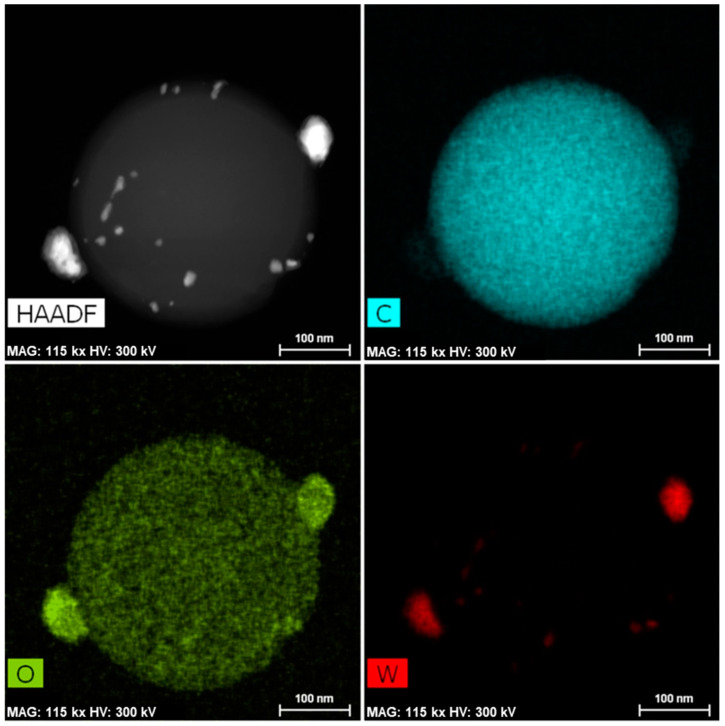
TEM-HAADF and EDX mapping images of CSW5.

The development of the microporosity and the surface area in the tungsten spheres is probably due to the gasifying action of said metal during carbonization [33]. Thus, a higher tungsten content should produce more gasification, as is clearly the case with the samples CSW2 and CSW5. However, the CSW10 sample, although clearly more porous than the CS, has not followed the suggested trend, which may be due to either an extremely small particle size of W that causes more attenuated gasification activity, or worse dispersion of the metallic phase on the surface of the sphere, so that the gassing activity in the carbonaceous phase would occur to a lesser extent. In any case, a notable part of the tungsten could be the porosity, as a consequence of the impregnation method, because the mesoporous volumes progressively decreased (Table 1). Regarding the morphology of these materials, the images of scanning electron microscopy (SEM) and transmission electron microscopy (TEM) clearly show that spheres with average diameter sizes between 300 and 400 nm have been obtained (Figure 2 and Figure 3), mostly isolated, although partially fused aggregates are also observed, and are more abundant in the case of the tungsten spheres than in the CS sample. The presence of tungsten carbide particles has been detected in the tungsten spheres, by SEM and EDX analysis (Figure 3 and Figure 4). The red mark in Figure 3d indicates the exact point of the EDX measurement. However, it was also found in the presence of tungsten oxide particles by TEM (Figure 5), whose existence has been corroborated by XPS.

**Figure 6:**
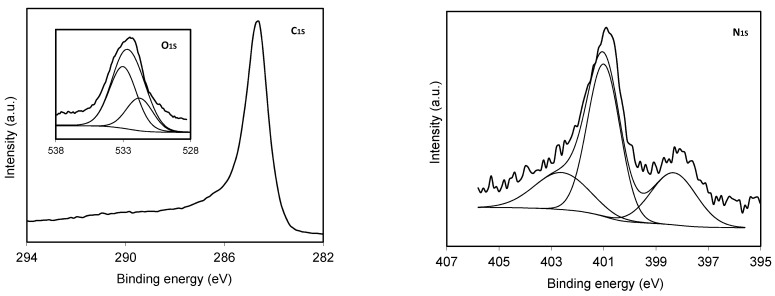
XPS spectra of the C_1S_, O_1S_ and N_1S_ regions for the CS sample.

Table 1, Table 2, Table 3, Table 4 and Table 5 show the chemical characterization data of the materials. The good adjustment of the metallic content obtained, with respect to the theoretically calculated value, should be noted (Table 1). On the other hand, the high content of superficial N in the CS sample is very remarkable, which reaches 7%, a relevant fact when taking into account the high temperature of carbonization (900 °C) and the thermal lability of this type of surface complex [34]. On the other hand, when the organic gel spheres impregnated with tungsten were carbonized, a decrease in both the oxygen and nitrogen content, relative to the CS material, was clearly observed, and in parallel to the gasification process. Even so, C spheres coated with tungsten also have a notable percentage of surface nitrogen, around 1.5% by weight (Table 2). This surface nitrogen is part of different functionalities or surface complexes of nitrogen (Table 3), type N-6 or pyridinic, type N-Q or quaternary, or type N-X, which would correspond to pyridine nitrogen bound to oxygenated groups [35]. The N-Q type groups are the most numerous in all the prepared materials, exceeding 50% of the total surface nitrogen in all cases (Table 3); meanwhile, the remaining nitrogen content is distributed into two equal parts between N-6 and N-X groups. Figure 6 shows the spectra of the C1S, O1S and N1S regions for CS electro-catalysts as an example. Both the C1S and O1S spectra are typical of well-carbonized carbon materials.

Finally, Table 4 shows the surface tungsten species detected by XPS, as well as the binding energies (B.E.) at which they have been detected. It should be noted that the W_XPS_ percentage is lower than the total obtained by TGA (Table 1) in the CSW2 and CSW5 samples, especially in the second one, which would indicate greater penetration of the metallic phase within the porous structure of the developed material during gasification. However, this phenomenon does not seem to be happening in the same way in the CSW10 sample, which could justify the surface area results described above.

**Figure 7:**
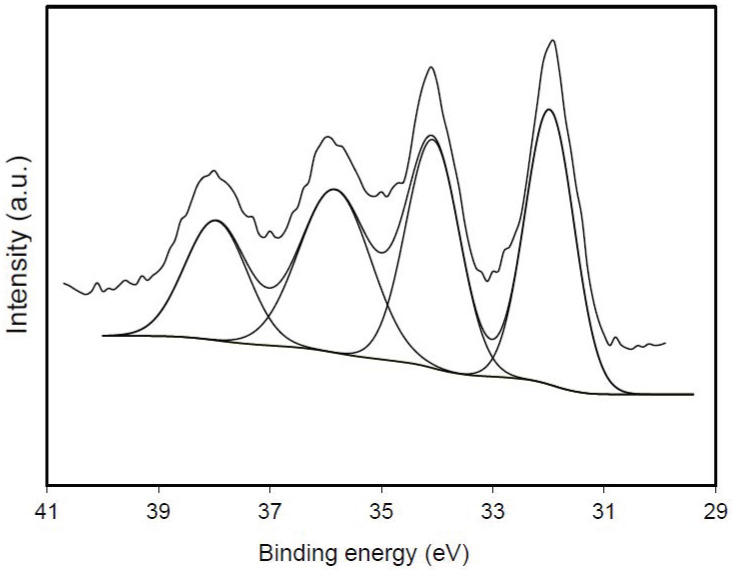
Deconvoluted XPS spectra of the W4f region for the CSW5 sample.

In any case, Table 4 clearly shows that all the materials doped with tungsten have WC and WO_3_ on their external surface, as the SEM-EDX and TEM-EDX analyses showed. However, the presence of WC gradually increases with the total content of tungsten, while that of WO_3_ decreases. Thus, while in the CSW2 sample, the presence of WC corresponds to 44% by weight of the total tungsten, in the sample CSW10, the presence of WC is clearly the majority, with 68%. As an example, Figure 7 shows the deconvolution of the W4f spectra of the sample CSW5.

**Figure 8:**
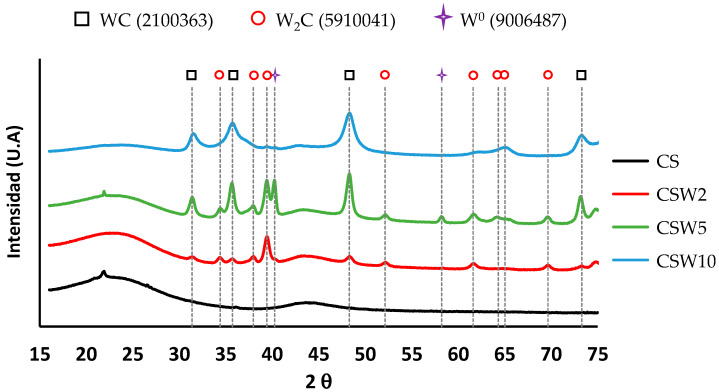
XRD patterns of all samples and the corresponding signal for WC, W_2_C and W^0^ phases.

Figure 8 shows the diffraction spectra of the materials. In all the tungsten-coated sphere samples, the presence of WC and W_2_C has been detected. In addition, there also appears to be W^0^ in the CSW5 sample. However, the formation of WO_3_ has not been detected in any sample, which suggests that this phase is composed of amorphous particles, or extremely small particles that are not diffracting; the formation of WO_3_ (detected by XPS) is related to tungsten oxidation when the samples are exposed to air, mainly from the W^0^ phase, since the carbide phases are more stable during spontaneous oxidation.

The average crystal sizes were obtained by applying the Scherrer equation to the diffraction data (Table 5).

**Figure 9:**
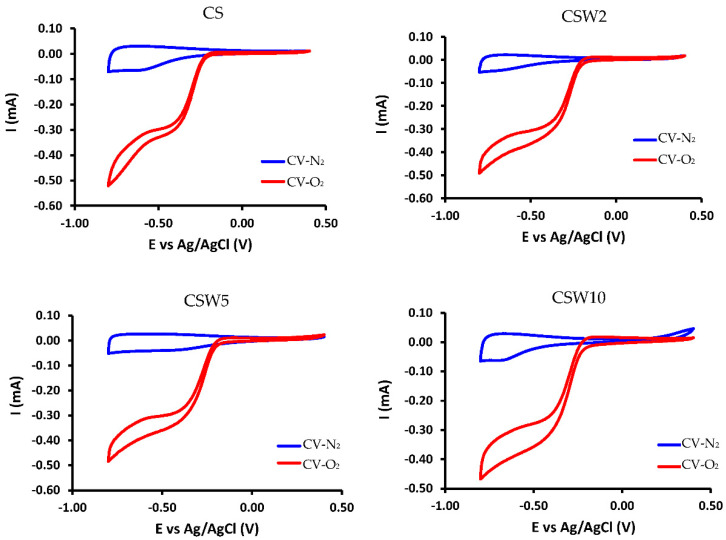
XRD cyclic voltagrams at 1000 rpm and 50 mV·s^−1^.

**Figure 10:**
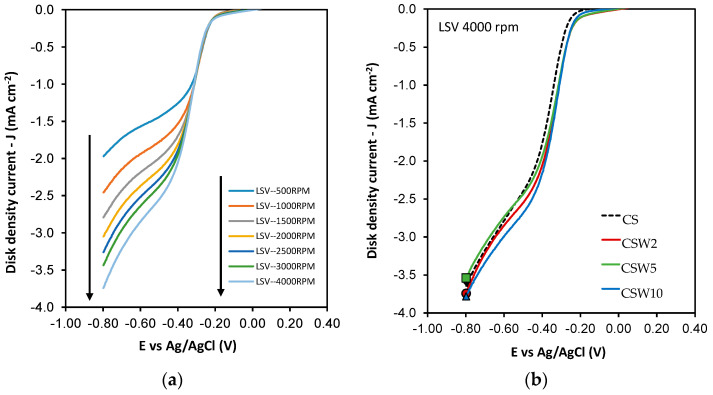
LSV curves for CS at different RDE rates (**a**), and LSV curves at 4000 rpm for all samples (**b**).

Figure 9 shows the cyclic voltammetries (CVs) performed in KOH 0.1 M, in the absence of oxygen (blue) and saturated in O_2_ (red), in which the activity of the electro-reduction of O_2_ is observed for all the samples. After the CVs, the LSV curves were obtained at different rotation speeds (Figure 10) for all the samples. Data from the LSVs were fitted to the Koutecky–Levich equation, and the parameters obtained are shown in Table 6.

All the calculated parameters, n, j_k,_ and E_ONSET,_ are shown in Table 6. As can be observed, all the samples catalyze the oxygen electro-reduction reaction through a mixed pathway of two and four electrons. However, the spheres with the highest nitrogen content (CS) tend, to a greater extent, to carry out electro-catalysis by the four-electron pathway. However, they need some optimization to advance via an electro-catalytic pathway exclusively. This improvement in activity for the CS material is due to the high nitrogen content, especially quaternary nitrogen [36]. The bibliographic results of two standard Pt/C catalysts, tested in similar experimental conditions to ours, have been included in Table 6. Both catalysts present good catalytic performance (n ≈ 4) with the lowest Eonset potentials; however, a high amount of Pt (20%) is loaded onto these samples. On the other hand, higher j_k_ values were obtained with our catalysts, despite the fact that they have very low metal loadings and they do not contain any noble metal.

The presence of tungsten on these spheres did not seem, a priori, to improve the electro-catalytic behavior. Nevertheless, it is worth mentioning that the nitrogen content in these spheres is five times lower than in the CS sample; therefore, a clear positive catalytic effect has taken place, which is in accordance with the results shown in Table 6. In addition, both the current and potential kinetic density values improve considerably with the presence of tungsten, despite significant nitrogen loss. However, the synergetic effect that the WC has with other electro-catalytic metals in this reaction cannot be easily evaluated with nitrogen in these materials, since both contents vary in opposite ways. In any case, these results are very interesting, showing that this strategy of combining tungsten and nitrogen on the carbon–nanosphere surface, as potential electro-catalysts, can work well, although they still need to be better optimized in order to shift the reduction mechanism to a pure four-electron pathway.

## 4. Conclusions

In the present work, carbon nanospheres were obtained by a simple solvothermal method. These spheres stand out for their high nitrogen content, despite the high carbonization temperature. In addition, during this carbonization process, tungsten carbide was formed, in those cases in which a tungsten precursor was added; thus, carbon spheres doped with nitrogen and coated with tungsten carbide were obtained.

The presence of tungsten during carbonization produced partial gasification of the spheres, favoring the development of the microporosity and the surface area.

All the prepared materials carried out the oxygen electro-reduction by a mixed pathway of two and four electrons, showing remarkable electro-catalytic behavior for the presence of both tungsten phases and nitrogen complexes.

## Figures and Tables

**Table 1:** Textural properties and total amount of W by TGA.

Sample	S_BET_ (m^2^·g^−1^)	V_0_ (cm^3^·g^−1^)	L_0_ (nm)	V_MESO_ (cm^3^·g^−1^)	W_TOTAL-TGA_ (%)
S	n.d.	-	-	-	0.0
CS	41	0.012	2.9	0.199	0.0
CSW2	254	0.107	2.7	0.191	2.7
CSW5	374	0.165	1.5	0.105	5.6
CSW10	96	0.043	2.4	0.087	10.0

**Table 2:** Surface chemical composition XPS.

Sample	% C_XPS_	% O_XPS_	% N_XPS_	% W_XPS_
CS	76.9	16.3	6.8	0.0
CSW2	92.3	4.0	1.5	2.2
CSW5	91.4	4.0	1.3	3.2
CSW10	83.4	4.9	1.6	10.1

**Table 3:** Surface content of nitrogen complexes obtained by XPS, with the corresponding binding.

Sample	% N_XPS_ Total	% N-6	% N-Q	% N-X
CS	6.8	25	55	22
CSW2	1.5	18	66	16
CSW5	1.3	23	57	20
CSW10	1.6	25	53	22
B.E. (eV)	N1s	398.5	401.0	402.5

**Table 4:** Surface tungsten content obtained by XPS. The percentage of both species are referred to the total.

Sample	% W_XPS_ Total	% WC	% WO_3_
CS	0.0	-	-
CSW2	2.2	44	56
CSW5	3.2	57	43
CSW10	10.1	68	32
B.E. (eV)	W4f_7/2_	32.0	35.7

**Table 5:** Average crystal size obtained by XRD.

Sample	d (nm)		
	WC	W_2_C	W^0^
CSW2	11.4	14.1	-
CSW5	12.2	13.6	18.1
CSW10	7.1	-	-

**Table 6:** Electrochemical parameters obtained from LSV curves obtained at −0.8 V.

Sample	n	j_K_ mA·cm^−2^	E_ONSET_ V
CS	3.51	6.31	−0.265
CSW2	3.19	7.14	−0.253
CSW5	3.09	6.60	−0.243
CSW10	3.17	7.28	−0.241
Pt/Vulcan (carbon black) [37]	4.00	5.00	-
Pt/C (graphitic carbon) [38]	3.90	5.00	−0.05

## Data Availability

The data presented in this study are available on request from the corresponding author.
